# Serum nitric oxide concentration in generalized chronic and aggressive periodontitis in the Mexican population is not related to the severity of the disease.

**DOI:** 10.7705/biomedica.6690

**Published:** 2023-03-30

**Authors:** Martha Graciela Fuentes-Lerma, Ana Lourdes Zamora-Pérez, Cecilia Robles-Gómez, Celia Guerrero-Velázquez, Jorge Peregrina-Sandoval, Melva Gutiérrez-Angulo, Rocío Patricia Mariaud-Schmidt

**Affiliations:** 1 Instituto de Investigación en Odontología, Centro Universitario de Ciencias de la Salud, Universidad de Guadalajara, Guadalajara, México Universidad de Guadalajara Universidad de Guadalajara Guadalajara Mexico; 2 Laboratorio de Inmunobiología, Departamento de Biología Molecular, Centro Universitario de Ciencias Biológico Agropecuarias, Universidad de Guadalajara, Guadalajara, México Universidad de Guadalajara Universidad de Guadalajara Guadalajara Mexico; 3 Departamento de Ciencias de la Salud, Centro Universitario de los Altos, Universidad de Guadalajara, Guadalajara, México Universidad de Guadalajara Universidad de Guadalajara Guadalajara Mexico

**Keywords:** periodontitis, nitric oxide, aggressive periodontitis, chronic periodontitis, alveolar bone loss, periodontitis, óxido nítrico, periodontitis agresiva, periodontitis crónica, pérdida de hueso alveolar

## Abstract

**Introduction:**

Periodontitis is an inflammatory disease that affects the supporting tissues of teeth, the effects of excess of nitric oxide, may contribute to the symptoms of periodontitis.

**Objective:**

To determine the serum nitric oxide concentration in generalized chronic and aggressive periodontitis patients and to compare it with a healthy subject group from the Mexican population.

**Materials and methods:**

A case and control study was performed. Sixty-nine individuals were recruited from the *Clínica de Posgrado de Periodoncia* of the *Centro Universitario de Ciencias de la Salud*, Universidad de Guadalajara, México. Patients with clinical features of generalized chronic periodontitis (GCP group, n=19), generalized aggressive periodontitis (GAP group, n=11), and a group of healthy subjects (HS group, n=39) were included in the study. Informed consent was obtained from each subject, and serum nitric oxide concentration was measured by an enzyme-linked immunosorbent assay.

**Results:**

Nitric oxide concentration in the study groups was greater in the GCP group (462.57 ± 16.57 µmol/L) than in the GAP group (433.84 ± 18.61 µmol/L) and the HS group (422.46 ± 12.07 µmol/L). A comparison using Student’s t-test (one-tailed) between healthy subjects and generalized chronic periodontitis showed borderline significance (p<0.04), whereas no significant differences were observed in HS and GAP groups, with a p-value of 0.64, and the GAP vs. GCP p-value was 0.33.

**Conclusion:**

The serum nitric oxide concentration observed in the present study suggests that nitric oxide plays a major role in the inflammatory process, which cannot necessarily be linked to the severity of the disease and periodontal tissue destruction.

## Resumen

**Introducción.:**

La periodontitis es una enfermedad inflamatoria que afecta los tejidos de soporte dental; los efectos del exceso de óxido nítrico pueden contribuir a los síntomas de la periodontitis.

**Objetivo.:**

Determinar la concentración de óxido nítrico en el suero de los pacientes con periodontitis agresiva y crónica generalizada, y compararla con la de individuos sanos de población mexicana.

**Materiales y métodos.:**

Se trata de un estudio de casos y controles. Se incluyeron 69 individuos de la Clínica de Posgrado de Periodoncia del Centro Universitario de Ciencias de la Salud de la Universidad de Guadalajara. Se dividieron en tres grupos: pacientes con periodontitis crónica generalizada (GCP, n=19), pacientes con periodontitis agresiva generalizada (GAP, n=11) e individuos sanos periodontalmente (HS, n=39). Se obtuvo el consentimiento informado de todos los participantes. Se utililizó la prueba ELISA para medir la concentración de óxido nítrico en suero.

**Resultados.:**

Las concentraciones de óxido nítrico observadas fueron mayores en el grupo GCP (462,57 ± 16,57 µmol/L) que en los grupos GAP (433,84 ± 18,61 µmol/L) y HS (422,46 ± 12,07 µmol/L). La comparación entre HS y GCP mediante la prueba estadística t de Student (una cola), mostró diferencias significativas (p<0,04), y no se observaron diferencias entre los grupos HS y GAP (p=0,64), ni entre GAP y GCP (p=0,33).

**Conclusiones.:**

La concentración de óxido nítrico en suero, observada en el presente estudio, sugiere que el óxido nítrico desempeña un importante papel en el proceso inflamatorio, lo que no necesariamente está ligado a la gravedad de la enfermedad ni a la destrucción del tejido periodontal.

Periodontal disease contributes significantly to the international burden of oral disease and is one of the most important dental diseases affecting human society at high prevalence rates ^1^. Seventy percent of the global population has presented with damage to periodontal supporting tissues ^2^. Periodontitis is an inflammatory disease that involves the supporting dental tissues, leading to the large destruction of connective tissue junctions and the alveolar bone ^3^^,^^4^.

Periodontitis is associated with gram-negative bacteria, including *Porphyromonas gingivalis*, *Prevotella intermedia* and *Aggregatibacter actinomycetemcomitans*, which trigger macrophages to produce nitric oxide (NO) and proinflammatory molecules such as interleukin-1 (IL-1) and tumor necrosis factor-α (TNF-α) ^5^^-^^7^.

Aggressive periodontitis affects adolescents and young adults. It shows an accelerated destruction of periodontal tissues with alveolar bone loss in otherwise clinically healthy subjects ^8^^,^^9^. In comparison, chronic periodontitis is the most frequent form of periodontitis and is characterized by the moderate evolution of periodontal attachment loss in older individuals ^10^.

Nitric oxide is now accepted as a prevalent biological mediator in many organisms. In mammals, nitric oxide is involved in several intercellular and intracellular activities, such as blood vessel dilation, neuronal intermediary, cytotoxicity, regulation of the cardiac rhythm and cellular respiration activities ^11^. Nitric oxide also acts as an important endogenous inhibitor of platelet and neutrophil aggregation and adherence to the normal endothelium ^12^.

Nitric oxide originates from a cluster of isoenzymes denominated nitric oxide synthases (NOS), which exist as three specific isoforms: endothelial NOS (eNOS), neural NOS (bNOS), and inducible NOS (iNOS). eNOS and bNOS deliver a limited amount of nitric oxide for a brief time following receptor stimulation. In contrast, iNOS is expressed due to proinflammatory mechanisms and generates a high volume of nitric oxide for longer periods.

The progression of chronic harmful inflammation of the periodontium may be a disrupted process. The effects of spare nitric oxide in the gingival mucosal tissue could contribute to the progression of the most frequent clinical signs of periodontitis in humans. The vasodilatory action of nitric oxide could be related to gingival redness, and gingival swelling may be provoked by the increase in the permeability of blood vessels induced by nitric oxide. The enlarged propensity of gingival tissue to bleed on probing may demonstrate the inhibitory mechanism of nitric oxide on platelet aggregation and the inhibitory activity of nitric oxide on adhesion ^13^.

A high concentration of nitric oxide produced locally is crucial in nonspecific host defense because of its cytotoxic activity against several organisms, as well as tumor cells. Nitric oxide released by eNOS plays a role in maintaining periodontal vascular perfusion ^13^.

Reher, *et al*., were the first to show that the salivary nitric oxide concentration is associated with the severity of periodontitis, allowing to differentiate between moderate and advanced chronic periodontitis. They proposed that NOS inhibitors could be valuable in the treatment of periodontal disease ^14^.

This study aimed to measure the serum nitric oxide concentration in patients with generalized chronic and aggressive periodontitis and compare it with that of healthy subjects.

## Material and methods

Sixty-nine individuals were recruited from the *Clínica de Posgrado de Periodoncia, Centro Universitario de Ciencias de la Salud*, Universidad de Guadalajara. Patients with clinical features of generalized chronic and aggressive periodontitis and healthy subjects were included in the study ^8^.

Medical and dental records were taken from all participants. None had a history of current smoking or systemic disease; or had received antibiotic, immunomodulatory, or anti-inflammatory drugs; or had received periodontal treatment within the previous six months. Pregnant and breastfeeding women were excluded from the study. The purpose of the study was explained fully to each participant before they were accepted into the study.

Institutional ethics review committee approval for the study was obtained (CI-01715), and informed consent was obtained from each participant in accordance with the 2013 Helsinki Declaration.

Clinical parameters (PD: probing depth, CAL: clinical attachment level, and %BoP: bleeding on probing) were measured at six sites per tooth (mesiobuccal, midbuccal, distobuccal, distolingual, midlingual, and mesiolingual) using a periodontal probe (15 mm, probe tip diameter 0.5 mm; University of North Carolina UNC-15 Hu-Friedy^®^ [Hu-Friedy, Chicago, IL, USA]) by a single researcher.

The individuals enrolled were classified according to the clinical and radiographic criteria proposed by the International World Workshop for a Classification of Periodontal Disease and Conditions 1999 ^8^.

### Generalized chronic periodontitis group

This study group included 19 patients (15 women and 4 men, aged 35 to 60 years old of age; mean age 48 ± 10 years). The amount of destruction was consistent with local factors, including subjects with ≥30% of sites with pockets, including a CAL of ≥5 mm and PD of ≥6 mm with radiographic evidence of alveolar bone loss ^8^.

### Generalized aggressive periodontitis group

This study group comprised 11 patients (10 women and 1 man ranging in age from 16 to 42 years of age; mean age 29 ± 6 years); they had a family history of ≥1 family member with severe periodontal damage. They showed radiographic evidence of severe alveolar bone loss and clinical attachment loss minimum of ≥5 mm in eight or more teeth, at least three of which were not central incisors or first molars, and PD ≥6 mm (8).

### Healthy subjects

This study group included 39 participants (22 women and 17 men ranging in age from 18 to 30 years of age; mean age 24 ± 7 years) without history of systemic diseases or tobacco smoking and health status (without clinical inflammation or BoP, with <3 mm of PD, and no evidence of CAL or radiographic bone loss) ^8^.

### Nitric oxide quantification

Venous blood from patients and healthy subjects was collected, and serum samples were separated by centrifugation at 900g (10 minutes). The obtained samples were stored and frozen immediately at -70 °C until analysis. Serum nitric oxide levels were measured by enzyme-linked immunosorbent assay, ELISA (R&D Systems, Minneapolis, MN, USA for total NO/Nitrite/ Nitrate ELISA kit, catalog number KGE001). Optical density was measured in a microplate reader set to 450 nm with wavelength correction at 540 nm. The concentrations were calculated with the standard curve included in each assay kit and were expressed as µmol/L.

### Statistical analysis

Clinical and demographic parameters in the study groups were determined by Student’s t-test and Fisher’s exact test, respectively. The nitric oxide concentration was compared among the three groups using Student’s t-test. Significance was considered at a p value <0.05.

## Results

### Demographic characteristics and periodontal clinical parameters

In terms of age, a difference was observed: the HS group was younger than the GCP group (p<0.0001); also, the comparison of the GCP vs. GAP groups showed a significant difference (p<0.0001). In terms of sex, women predominated in all the study groups and did not exhibit differences.

Among the periodontal clinical parameters, PD demonstrated statistical significance between the HS vs. GCP and HS vs. GAP groups (p<0.0001) in both cases when using Student’s t test (table 1).

The serum nitric oxide concentrations are presented as the means ± standard deviations. The nitric oxide concentration did not differ significantly between the GAP group (433.84 ± 18.61 µmol/L) and the GCP group (462.57 ± 16.57 µmol/L) or between the GAP group and the HS group (422.46 ± 12.07 µmol/L). The comparative analysis was only significant between GCP and HS, but it showed borderline significance (p=0.04) (table 1).

**Table 1:** Clinical parameters and serum nitric oxide concentration in study groups

	HS (n=39)	GCP (n=19)	GAP (n=11)	HS vs. GCP p value	HS vs. GAP p value	GCP vs. GAP p value
Age (years) (mean ± SD)	24 ± 7	48 ± 10	29 ± 6	0.00*	0.03*	0.00*
Female (n)	22	15	10	0.14**	0.07**	0.62**
Male (n)	17	4	1	-	-	-
PD (mm)	1.02 ± 0.32	4.02 ± 1.02	4.30 ± 1.12	0.00*	0.00*	0.49*
CAL (mm)	0	4.65 ± 1.59	4.89 ± 1.55	-	-	0.69*
BoP (%)	0	100	100	-	-	-
Serum nitric oxide concentration (mean ± SD)	422.46 ± 12.07	462.57 ±1 6.57	433.84 ± 18.61	0.04*	0.64*	0.33*

Significantly different p<0.05; HS: healthy subject group; GCP: chronic periodontitis; GAP: generalized aggressive periodontitis; PD: probing depth; CAL: clinical attachment level; BoP: bleeding on probing

* t Student (one tailed)

** Fisher exact test

## Discussion

The participation of nitric oxide in physiological actions depends on its origin, duration, and concentration ^15^. Moreover, as Wadhwa, *et al*. ^16^, concluded that nitric oxide levels in saliva and serum are positive clues to estimate the illness condition of periodontal tissues ^16^, and as Schmidt, *et al*. ^17^, described that nitric oxide also exhibits anti-inflammatory effects. It plausibly acts in two pathways, where decreased levels inhibit, and large concentrations worsen the inflammatory process ^17^. In addition, Wang, *et al*. ^18^, found that the nitric oxide present in the serum substantially increased with periodontitis evolution (p<0.00) in a rat model of periodontitis ^18^.

Contrary to Schmidt, *et al*. (17), and Wang, *et al*. ^18^, in our study, we observed high concentrations of nitric oxide in the GCP group compared to the HS group (p=0.04), which demonstrated a moderate pattern of periodontal tissue destruction compared to the GAP group, which exhibited severe periodontal destruction and alveolar bone loss ^8^. Reher, *et al*. ^14^, found that nitric oxide concentrations in saliva are higher in patients with chronic periodontitis than in healthy people and are related to the severity of the disease. Interestingly, Scarel-Caminaga, *et al*., reported higher levels of salivary nitric oxide in periodontally healthy subjects than in chronic periodontitis patients ^19^.

In contrast to Reher, *et al*. ^14^, and Scarel-Caminaga, *et al*. ^19^, in the present study, we found differences between nitric oxide concentrations in the serum of GCP patients and the HS group (p=0.04) but not in the GAP and HS groups or between GCP and GAP; this behavior suggests that the severity of the disease is not related to the nitric oxide concentration in serum. Nevertheless, at decreased levels, nitric oxide exhibits cytoprotective functions ^15^ and may be essential for normal osteoclast activity ^20^; meanwhile, at increased concentrations, nitric oxide provokes cytotoxic effects in cancer cells and induces apoptosis ^15^^,^^21^.

The differences in nitric oxide concentration in GCP vs. HS could be explained by the fact that nitric oxide participates as a biomessenger of bone loss induced by inflammation ^22^. Sakurai, *et al*. ^23^, illustrated that nitric oxide might participate as a mediator of connective tissue destruction in arthritis (21), an inflammatory disease. This was also observed by Hukkanen, *et al*. ^24^, in metabolic bone and inflammatory diseases, where an imbalance between bone deposition and resorption provokes a loss of bone tissue related to postmenopausal osteoporosis, Paget’s disease, rheumatoid arthritis, and periodontal disease.

With respect to the origin of nitric oxide, McCarty, *et al*. ^25^, described that a moderate concentration of nitric oxide produced by eNOS plays a vital physiological role in maintaining bone density by stimulating new bone formation (25). Additionally, MacIntyre, *et al*. (26), noted that high nitric oxide production by endothelial cells in bone plays a physiological role by regulating the activity of osteoclasts, which probably limited the alveolar bone damage observed in the GCP group in the present study.

On the other hand, Daghigh, *et al*. ^27^, found that nitric oxide and iNOS concentrations are higher in fibroblasts from gingival tissue in patients with periodontitis than in cells of healthy subjects ^27^, and Sun, *et al*. ^28^, described higher iNOS levels in gingival tissue in rats with periodontitis than in the control group. In this regard, Lappin, *et al*. ^29^, and Batista, *et al*. ^30^, concluded that the iNOS concentration is elevated in biopsy tissue from patients with periodontitis compared with healthy tissue. Interestingly, Fukada, *et al*. ^31^, described that in iNOS-/- mice with experimental periodontitis, nitric oxide deficiency is related to an imbalance in osseous tissue resorptionmodulating factors, which trigger serious bone loss when stimulated by disease ^31^. Likewise, iNOS activation by cytokines inhibits the function of osteoblasts *in vitro* and stimulates osteoblast apoptosis ^32^.

The involvement of nitric oxide and NOS in apposition and bone resorption is controversial. Fukada, *et al*. ^31^, and Ralston, *et al*. ^33^, noted that decreased nitric oxide production by iNOS causes bone resorption, although Lowik, *et al*. ^34^, and Herrera, *et al*. ^35^, reported that iNOS inhibits bone resorption. In contrast, Armour, *et al*. ^36^, reported that eNOS is fundamental for differentiation and osteoblast activity and that its failure is related to decreased bone mass.

Additionally, MacIntyre, *et al*. ^26^, noted that the increase in nitric oxide production by endothelial cells (eNOS) inhibits bone resorption. In this regard, we did not observe differences in serum nitric oxide concentration among the GAP vs. HS and GAP vs. GCP groups. Although aggressive periodontitis has been described as a severe destruction pattern of alveolar bone in a short time ^8^, the lack of significant differences between these groups could be related to other risk factors, such as genetics involved in aggressive periodontitis development. Familial aggregation might be an interesting topic to explore, since a few genes have been described as important players involved in aggressive periodontitis in several populations, such as NOD2 ^37^^,^^38^.

Our results allow us to infer that iNOS and eNOS, along with other mechanisms or related metabolic pathways, probably account for the severity of alveolar bone loss in periodontitis. It is likely that the pathways that activate NOS, which produces nitric oxide, differ between generalized aggressive and chronic periodontitis, and in healthy subjects; the difference in pathways is probably related to the damage observed in these patients. Additionally, time-dependent effects and concentrations could participate in the phenotypic consequences of periodontitis.

Supplementary studies are required to address the role of nitric oxide in periodontitis. The higher serum nitric oxide concentration in patients in the GCP group suggests that nitric oxide plays a major and selective role in the inflammatory process and that the nitric oxide concentration and origin are probably not related to the severity of the disease.

## References

[1] Petersen PE, Ogawa H. (2005). Strengthening the prevention of periodontal disease: The WHO Approach. J Periodontol.

[2] Oppermann RV, Haas AN, Rosing CK, Susin C. (2015). Epidemiology of periodontal diseases in adults from Latin America. Periodontol 2000.

[3] Balwant R, Simmi K, Rajnish J, Suresh C. (2008). Biomarkers of periodontitis in oral fluids. J Oral Sci.

[4] Papapanou PN, Sanz M, Budunelli N, Dietrich T, Feres M, Fine DH (2018). Periodontitis: Consensus report of workgroup 2 of the 2017 world workshop on the classification of periodontal and peri-implant diseases and conditions. J Periodontol..

[5] Skaleric U, Gaspirc B, McCartney-Francis N, Masera A, Wahl S. (2006). Proinflammatory and antimicrobial nitric oxide in gingival fluid of diabetic patients with periodontal disease. Infect Immun.

[6] Norskov-Lauritsen N, Kilian M. (2006). Reclassification of Actinobacillus actinomycetemcomitans, Haemophilus aphrophilus, Haemophilus paraphrophilus and Haemophilus segnis as Aggregatibacter actinomycetemcomitans gen. nov., comb. nov., Aggregatibacter aphrophilus comb. nov. and Aggregatibacter segnis comb. nov., and emended description of Aggregatibacter aphrophilus to include V factor-dependent and V factor-independent isolates. Int J Sys Evol Microbiol.

[7] Okada H, Murakami S. (1998). Cytokine expression in periodontal health and disease. Crit Rev Oral Biol Med.

[8] Armitage G. (1999). Development of a classification system for periodontal diseases and conditions. Ann Periodontol.

[9] Tonetti M, Mombelli A. (1999). Early-onset periodontitis. Ann Periodontol.

[10] Flemmig T, Lindhe J, Niklaus L. (1999). Periodontitis. Ann Periodontol.

[11] Groves J, Wang C. (2000). Nitric oxide synthase: Models and mechanisms. Curr Opin Chem Biol.

[12] Stangl K, Cascorbi I, Laule M, Klein T, Stangl V, Rost S (2000). High CA repeat numbers in intron 13 of the endothelial nitric oxide synthase gene and increased risk of coronary artery disease. Pharmacogenetics.

[13] Lohinai Z, Szabó C. (1998). Role of nitric oxide in physiology and pathophysiology of periodontal tissues. Med Sci Monit.

[14] Reher V, Zenóbio E, Costa F, Reher P, Soares R. (2007). Nitric oxide levels in saliva increase with severity of chronic periodontitis. J Oral Sci.

[15] Choudhari S, Chaudhary M, Bagde S, Gadbail A, Joshi V. (2013). Nitric oxide and cancer: A review. World J Surg Oncol.

[16] Wadhwa D, Bey A, Hasija M, Moin S, Kumar A, Aman S (2013). Determination of levels of nitric oxide in smoker and nonsmoker patients with chronic periodontitis. J Periodontal Implant Sci.

[17] Schmidt H, Nau H, Wittfoht W, Gerlach J, Prescher K, Klein M (1988). Arginine is a physiological precursor of endothelium-derived nitric oxide. Eur J Pharmacol.

[18] Wang Y, Huang X, He F. (2019). Mechanism and role of nitric oxide signaling in periodontitis. Exp Ther Med.

[19] Scarel-Caminaga RM, Cera FF, Pigossi SC, Finoti LS, Kim YJ, Viana AC (2017). Inducible nitric oxide synthase polymorphisms and nitric oxide levels in individuals with chronic periodontitis. Int J Mol Sci.

[20] Brandi M, Hukkanen M, Umeda T, Moradi-Bidhendi N, Bianchi S, Gross S (1995). Bidirectional regulation of osteoclast function by nitric oxide synthase isoforms. Proc Natl Acad Sci USA.

[21] Brennan P, Thomas G, Langdon J. (2003). The role of nitric oxide in oral diseases. Arch Oral Biol.

[22] Grabowski P, England A, Dykhuizen R, Copland M, Benjamin N, Reid DM (1996). Elevated nitric oxide production in rheumatoid arthritis: Detection using the fasting urinary nitrate: Creatinine ratio. Arthritis Rheum.

[23] Sakurai H, Koshaka H, Lui M, Higashiyama H, Hirata Y, Kanno K (1995). Nitric oxide production and inducible nitric oxide synthase expression in inflammatory arthritides. J Clin Invest.

[24] Hukkanen M, Hughes F, Buttery L, Gross S, Evans T, Seddon S (1995). Cytokine-stimulated expression of inducible nitric oxide synthase by mouse, rat and human osteoblast-like cells and its functional role in osteoblast metabolic activity. Endocrinology.

[25] McCarty M. (2005). Supplemental arginine and high dose folate may promote bone health by supporting the activity of endothelial-type nitric oxide synthase in bone. Med Hypotheses.

[26] MacIntyre I, Zaidi M, Toehidul A, Datta H, Moonga B, Lidbury P (1991). Osteoclastic inhibition: An action of nitric oxide not mediated by cyclic GMP. Proc Natl Acad Sci USA.

[27] Daghigh F, Borghaei R, Thornton R, Bee J. (2002). Human gingival fibroblasts produce nitric oxide in response to proinflammatory cytokines. J Periodontol.

[28] Sun S, Zhang D, Wu Y, Yan L, Liu J, Pan C (2020). The expression of the inducible nitric oxide synthase in the gingiva of rats with periodontitis and diabetes mellitus. Arch Oral Biol.

[29] Lappin D, Kjeldsen M, Sander L, Kinane D. (2000). Inducible nitric oxide synthase expression in periodontitis. J Periodontal Res.

[30] Batista A, Silva T, Chun J, Lara V. (2002). Nitric oxide synthesis and severity of human periodontal disease. Oral Dis.

[31] Fukada S, Silva T, Saconato I, Garlet G, Ávila-Campos M, Silva J (2008). iNOS-derived nitric oxide modulates infection-stimulated bone loss. J Dent Res.

[32] Damoulis P, Hauschka P. (1997). Nitric oxide acts in conjunction with pro-inflammatory cytokines to promote cell death in osteoblasts. J Bone Miner Res.

[33] Ralston S, Ho L, Helfrich M, Grabowski P, Johnston P, Benjamin N. (1995). Nitric oxide: A cytokine- induced regulator of bone resorption. J Bone Miner Res.

[34] Lowik C, Nibbering P, van de Ruit M, Papapoulos S. (1994). Inducible production of nitric oxide in osteoblast-like cells and in fetal mouse bone explants is associated with suppression of osteoclastic bone resorption. J Clin Invest.

[35] Herrera BS, Martins-Porto R, Maia-Dantas A, Campi P, Spolidorio LC, Costa SKP (2011). iNOS-derived nitric oxide stimulates osteoclast activity and alveolar bone loss in ligature-induced periodontitis in rats. J Periodontol.

[36] Armour K, Armour K, Gallagher M, Godecke A, Helfrich M, Reid D (2001). Defective bone formation and anabolic response to exogenous estrogen in mice with targeted disruption of endothelial nitric oxide synthase. Endocrinology.

[37] Sudo T, Okada Y, Ozaki K, Urayama K, Kanai M, Kobayashi H (2017). Association of NOD2 mutations with aggressive periodontitis. J Dent Res.

[38] Mizuno N, Kume K, Nagatani Y, Matsuda S, Iwata T, Ouhara K (2020). Aggressive periodontitis and NOD2 variants. J Hum Genet.

